# Phytochemical Screening and Antidiabetic Potential of *Paeonia emodi* in Alloxan‐Induced Diabetic Rats: Investigative Study Toward Possible Mechanism

**DOI:** 10.1002/fsn3.70870

**Published:** 2025-09-01

**Authors:** Jirui Fan, Muhammad Ibrar, Mir Azam Khan, Abdullah, Muhammad Saeed Jan, Madeeha Shabnam, Maqsood ur Rehman

**Affiliations:** ^1^ Department of Pharmacy Heilongjiang Eye Hospital, Harbin China; ^2^ Department of Pharmacy Bacha Khan University Charsadda Pakistan; ^3^ Department of Pharmacy University of Malakand Chakdara Pakistan; ^4^ Department of Chemistry Women University Mardan Mardan KP Pakistan

**Keywords:** α‐amylase, α‐glucosidase, antioxidant, histopathological studies, liver function tests, *P. emodi*, serum insulin

## Abstract

*Paeonia emodi* has long been used in folk medicine to treat a variety of ailments, including diabetes, skin disorders, dropsy, cuts, wounds, ulcers, fever, and blood disorders, etc., which are generally categorized under the complications of diabetes mellitus. Various species of this genus have also been verified to possess strong anti‐diabetic activity. In this context, the current study was designed to investigate the antidiabetic potential to confirm the purported traditional use of *P. emodi* . Different solvent fractions of *P. emodi,* i.e., crude methanolic extract (Pe.Cr), n‐hexane (Pe.Hex), chloroform (Pe.Chf), ethyl acetate (Pe.EtAc), butanol (Pe.Bt) and aqueous (Pe.Aq) were used for in vitro studies against α‐glucosidase, α‐ amylase, dipeptidyl peptidase‐4 (DPP‐4), and protein tyrosine phosphatase 1B (PTP‐1B) using a spectrophotometer and microplate reader. Among all fractions, Pe.Bt had the most prominent activity and was subjected to GC–MS analysis, in vivo anti‐diabetic, pancreas protective, and hepatoprotective studies in alloxan‐induced diabetes rats. The animals administered with alloxan (except group1) having FBGL higher than 220 mg/dL were selected and placed in different groups. The first group, which served as a normal control, received normal saline. The second group received a 5% tween‐80 suspension, which served as diabetic control. The 3rd, 4th and 5th groups were administered Pe.Bt at the doses of 150, 300, and 500 mg/kg body weight, respectively, through oral gavage. Group six received metformin 50 mg/kg and served as the standard group. Fasting Blood glucose level (FBGL) was checked for 21 days. Liver function tests, insulin level, hepatoprotection, and pancreas protection study were investigated. The α‐glucosidase was inhibited effectively by Pe.Bt with an IC_50_ value of 626 μg/mL. The antioxidant activities also substantiated the excellent inhibitory potential of Pe.Bt. The IC_50_ value of Pe.Bt against DPPH and ABTS was figured out to be 77 and 139 μg/mL, respectively, which was relatively comparable with ascorbic acid. Similarly, Pe.Bt was found active against PTP‐1B and showed a moderate effect against DPP4 with IC_50_ of 45.13 and 92.45, respectively. In the time frame of 21 days, the metformin and Pe.Bt significantly decreased FBGL on the 21st day to 108 and 96 mg/dL, respectively. Similarly, rats treated with Pe.Bt (500 mg/kg) significantly lowered the level of alanine transaminase (ALT), aspartate aminotransferase (AST), alkaline phosphatase (ALP) and increased total protein level (TP) in comparison with the diabetic control group. The histopathological study revealed that rats treated with Pe.Bt (500 mg/kg) have a remarkable ameliorative effect on liver and pancreas histological architecture as compared to the diabetic control group. The findings of the current investigations indicated that Pe.Bt possesses strong anti‐diabetic, hepatoprotective, anti‐oxidant properties and alleviates alloxan‐induced pancreatic damage in diabetic rats.

## Graphical abstract

The findings of the current investigations indicated that the Butanol fraction obtained from *Paeonia emodi* possesses strong anti‐diabetic, pancreatic protective, hepatoprotective, anti‐oxidant properties and alleviates Alloxan‐induced liver and pancreatic damage in diabetic rats.

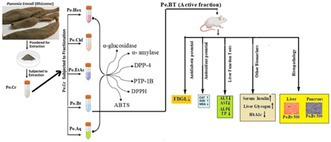

## 1 Introduction

The modern world is in a continuous struggle to fight for the survival of human beings. The battle for survival is against various factors, of which the most prominent one is “disease” (Wagner 2000). Multiple diseases have been proven to hinder the harmony of life on Earth in various ways. Among the most attention‐seeking health problems, diabetes mellitus (DM) (Jones et al. 2017), a chronic metabolic disorder (Duman et al. 2025), is considered one of the most rapidly growing and overwhelming diseases related to free radical generation. According to an International Diabetes Federation (IDF) report, elevated blood glucose is the third‐highest risk factor for premature mortality, following high blood pressure and tobacco use globally (Atlas 2015). In 2015, according to an IDF report, 415 million (8.8%) adults (aged 20–79) worldwide were estimated to have diabetes; this number is expected to rise to 642 million (10.4%) by 2040, or one adult in ten (Zhang et al. 2021). DM is associated with numerous complications, including retinopathy, neuropathy, nephropathy, and vascular insufficiency (Aktas 2023); (de Groot et al. 2001). There are myriad etiological factors for DM, but oxidative stress is considered the key factor for this disease. Overproduction of reactive oxygen species (ROS) can damage DNA, lipids, proteins, and other components of cells, causing cellular malfunction and disruption of normal physiological processes. This damage has the potential to cause inflammation and impair the function of vital cell components, which in turn contributes to the development and progression of many diseases, such as diabetes (Evans et al. 2002). Oxidative stress may damage the β‐cells of the pancreas and instigate the hyperglycemic conditions (Betteridge 2000). The liver is recognized as the primary metabolic organ of the body, and a defect in the liver may lead to various diseases (Kosekli and Aktas 2025). It plays a key role in preserving normal glucose levels through different mechanisms, and it serves as the primary location for insulin clearance (Eidi and Eidi 2009). Diabetes is now considered the leading cause of liver disorders worldwide, accompanied by obesity, cardiovascular disorders, hyperglycemia, and dyslipidemia (Basaran and Aktas 2025). The major causes of mortality in diabetic patients are abnormalities in enzyme levels, carcinoma of hepatocytes, and acute liver failure. There are several treatment techniques available, including insulin, natural and synthetic drugs, and, particularly, cellular therapy, but each has its limitations (Pandit et al. 2010). The focus of advanced research teams in pharmaceutical sciences is diverting towards novel sources of medicines that make it possible for each and every individual to get access to economic, safe, and effective chemotherapy (Cragg and Newman 2013). Natural medicine is never disregarded in terms of economy, safety, and effectiveness (Newman and Cragg 2012). Several plants have been reported to possess strong anti‐diabetic potential. Several bioactive compounds with anti‐diabetic properties have been isolated from various plant species (Coman et al. 2012). Numerous plants have also been verified to possess a postprandial hypoglycemic effect through different mechanisms, i.e., modulating gastrointestinal motility (especially slowing gastric emptying) and stimulating the incretin system are major targets for the management of postprandial glycemia (Roman‐Ramos et al. 1995; Kamruzzaman et al. 2021). The postprandial hypoglycemic effect of a chemical compound is due to the inhibition of α‐glucosidase, which is responsible for the production of glucose molecules in the intestine (Ag 1994). Similarly, several plant species have also been verified to possess antioxidant compounds, which have a major role in the prevention of DM. The antioxidant compounds identified in various plant species usually belong to a group of secondary metabolites called flavonoids. Medicinal plants are usually rich in this group of secondary metabolites (Babu et al. 2013).

*Paeonia emodi* belongs to the family Paeoniaceae. This family consists of the only medicinally important genus, *Paeonia*. Almost all the species of this genus have been scientifically verified to possess considerable amounts of flavonoids and phenolic compounds (Kamiya et al. 1997; He et al. 2010). *P. emodi* has been reported for various pharmacological activities like antifungal, antibacterial, insecticidal, phytotoxic, cytotoxic, antioxidant, urease inhibition, α‐chymotrypsin, and lipoxygenase inhibitory activities (Khan et al. 2005; Ismail et al. 2003; Riaz et al. 2004). It is also used for the treatment of headache, hysteria, abdominal spasms, diarrhea, dysentery, vomiting, constipation, respiratory diseases, and epilepsy. Besides these disorders, this plant has been folklorically used for cardiac diseases like hypertension, palpitations, congestive heart failure, and atherosclerosis (Ghayur et al. 2008; Zargar et al. 2014).

Similarly, numerous species of this genus have also been discovered to have anti‐diabetic properties and have a beneficial effect against diabetic nephropathy (Hsu et al. 1997; Kishore et al. 2017). The phytochemical screening of *P. emodi* has been reported with sufficient quantities of flavonoids, alkaloids, phenols, tannins (Uddin et al. 2013; Ali et al. 2024) and is reported for various biological activities. This plant has also importance in the context of ethnomedicine and is used traditionally for the treatment of skin disorders, dropsy, cuts, wounds, ulcers, fever, cardiovascular diseases, and diabetes mellitus (Alamgeer et al. 2013; Uniyal and Shiva 2005; Tiwari et al. 2010; Ibrar et al. 2019; Rana 2013). Based on the traditional importance and literature review, the current project is designed to evaluate the anti‐diabetic and hepatoprotective potential of *P. emodi* in Alloxan‐induced diabetic rat models. The study focuses on assessing glycemic control, liver enzyme levels (ALT, AST, ALP), lipid profile, insulin level, and oxidative stress markers (such as MDA, SOD, and CAT) to determine the therapeutic efficacy of *P. emodi* . It is hypothesized that treatment with *P. emodi* extract will significantly reduce blood glucose levels, improve liver function, and enhance antioxidant status in diabetic rats compared to the untreated diabetic control group. This directional hypothesis is based on the reported flavonoid and phenolic content of *P. emodi* , which are known to exert anti‐diabetic and hepatoprotective effects.

## 2 Materials and Methods

### 2.1 Plant Collection and Extraction

The plant was collected in July 2023 and authenticated by Dr. Nasrullah, Department of Botany, University of Malakand. The voucher specimen was submitted to the herbarium of the same institute under reference number H.UOM.BG.226. The rhizomes were separated from the plant and washed with distilled water to remove soil and dust. They were converted into small pieces and shade dried for 30 days. Dried rhizomes weighing 4 kg were converted into coarse powder with a cutter mill, followed by maceration for 15 days in 80% methanol in a large container. The mixture was filtered, and the filtrate obtained was subjected to evaporation using a rotary evaporator (Heidolph Laborota 4000, Schwabach, Germany) at 40°C. This resulted in 310 g (7.75%) of brownish semisolid crude methanolic extract (Pe.Cr). The Pe.Cr weighing 250 g was subjected to fractionation through a successive solvent extraction process with increasing polarity (Ibrar et al. 2018; Ahmad et al. 2015). Similarly, the solvent fractions obtained through the separating funnel were Pe.Hex, Pe.Chf, Pe.EtAc, Pe.Bt, and Pe.Aq weighing 62 g (24.8%), 19 g (7.6%), 32 g (12.8%), 29 g (11.6%) and 96 g (38.4%) respectively.

### 2.2 In‐Vitro α‐Glucosidase Inhibitory Activity

The α‐glucosidase inhibitory potential of various samples of *P. emodi* (Pe.Hex, Pe.Chf, Pe.EtAc, Pe.Bt, Pe.Aq) was carried out following the standard procedure (McCue et al. 2005). Briefly, the enzyme, i.e., α‐glucosidase solution, was prepared by dissolving 0.5 units/mL in a 0.1 M phosphate buffer having a pH of 6.9. Similarly, the substrate solution, i.e., *p*‐Nitrophenyl‐α‐D‐glucopyranoside (5 mM), was prepared in the same buffer solution. Likewise, the test samples had concentrations of 1000, 500, 250, and 125 μg/mL of plant samples, including Pe.Hex, Pe.Chf, Pe.EtAc, Pe.Bt, and Pe.Aq, prepared, were properly mixed with α‐glucosidase solution and incubated at 37°C for 15 min. After incubation, 20 μL of substrate was transferred to each test tube of the enzyme. By adding 80 μL of 0.2 M sodium carbonate solution, the reaction was completed. Finally, the absorption value of each sample was recorded at 405 nm using a double beam UV spectrophotometer. The mixture having no α‐glucosidase was used as a blank, and the mixture having acarbose as a test sample served as a positive control. Each experiment was performed in triplicate, and the percentage of inhibition was calculated using a formula.

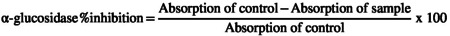




### 2.3 α‐ Amylase Inhibitory Activity

Alpha amylase assay evaluation was carried out by following standardized reported protocols (Huneif et al. 2022). Phosphate buffer and the enzyme were combined to prepare the enzymatic solution, to which test samples of various potencies were later added. The sample mixture was then incubated, and the test and control solutions' additions of starch and dinitro salicylic acid solution followed. A microplate reader was used to measure the absorbance at 656 nm after the mixture was briefly placed in a boiling water bath.

The following formula was used to calculate the α‐amylase enzyme's % inhibition potential.

$$\alpha ‐\text{amylase}\;\%\text{inhibition}=\frac{\text{Absorption of control}–\text{Absorption of sample}}{\text{Absorption of control}}\;x\;100$$

### 2.4 Dipeptidyl Peptidase‐4 (DPP‐4) Assay

A fluorescent probe‐based in vitro test was used by Quek et al. (2021) to evaluate the inhibitory effect of several *P. emodi* samples (Pe.Hex, Pe.Chf, Pe.EtAc, Pe.Bt, and Pe.Aq) on the DPP‐4 target. The assay was carried out in a 96‐well microplate reader, and the tested samples were incubated with recombinant human DPP‐4 and tetraphenylethene‐lys‐Phe‐Pro‐Glu (TPE‐KFPE) in HEPES buffer at 37°C for 30 min. The compound's activity was first assessed at various doses, and then a dose‐dependent response was used to estimate the IC_50_ value following standard procedure.

### 2.5 Protein Tyrosine Phosphatase 1B (PTP1B) Test

The protein tyrosine phosphatase 1B in vitro assay on various *P. emodi* samples (Pe.Hex, Pe.Chf, Pe.EtAc, Pe.Bt, and Pe.Aq) was carried out as per the reported method (Huneif et al. 2022). In order to create solutions of p‐NO_2_ ‐phenol phosphate (1 mM), PTP1B (10 mM), and different quantities of the substance, a 3,3‐dimethyl glutarate buffer solution with a pH of 7 was used. The solutions were incubated for 40 min at 27°C, and then the absorbance at 405 nm was measured. The trials were conducted three times, and the compound's IC_50_ was determined using standard procedures.

### 2.6 GC–MS Analysis

The fraction revealing strong potential against enzymes responsible for diabetes was subjected to GC–MS analysis for identification of bioactive chemicals (Ezhilan and Neelamegam 2012). The chemical compounds in the Pe.BT were identified by comparing them with existing compounds described in the literature based on the retention time. Furthermore, spectral data from the NIST and Wiley libraries were utilized for the validation of these compounds.

### 2.7 DPPH Radical Scavenging Assay

In this assay, various samples of *P. emodi* were screened against 1,1‐diphenyl, 2‐picrylhydrazyl (DPPH) free radicals following the standard reported procedure (Ahmad et al. 2016). Different concentrations, i.e., 125, 250, 500, and 1000 μg/mL, of plant samples including Pe.Hex, Pe.Chf, Pe.EtAc, Pe.Bt, and Pe.Aq were used in this study. Plant sample (0.1 mL) was added to 1 mL of DPPH solution (0.004% in methanol). After incubation for 30 min, the absorbance was determined at 517 nm using a double beam spectrophotometer. Ascorbic acid was employed as a positive control. The following equation was used to calculate the percent radical scavenging potential.

$$\left[\left(A–B\right)/A\right]\times 100$$

where *A* denotes control absorbance and *B* represents the absorbance of test samples.

### 2.8 ABTS Free Radical Scavenging Activity

The antioxidant activity was carried out against 2,2‐azinobis [3‐ethylbenzthiazoline]‐6‐sulfonic acid (ABTS) as a free radical (Ayaz et al. 2014). Briefly, solutions of ABTS (7 mM) and potassium persulphate (K_2_S_2_O_4_) 2.45 mM were mixed properly and kept overnight in the dark at 25°C to obtain a colored solution. The working solution of ABTS with an absorbance of 0.70 at 734 nm was obtained by diluting the ABTS solution with phosphate buffer (0.01 M) having pH 7.4. The test samples (Pe.Hex, Pe.Chf, Pe.EtAc, Pe.Bt, Pe.Aq) with various concentrations (1000, 500, 250, 125 g/mL) were added to 3 mL of ABTS solution. After a 15‐min incubation period, the absorbance of each sample was measured at 734 nm with a double beam spectrophotometer. In this assay, ascorbic acid was used as a positive control. The percent radical scavenging was obtained using the equation:

$$\%\text{scavenging effect}=\left[\left(A–B\right)/A\right]\times 100$$

where *A* denotes control absorbance and *B* represents the absorbance of test samples.

### 2.9 In Vivo Anti‐Diabetic Assay

In vivo anti‐diabetic activity was only conducted for the most active fraction of *P. emodi* as follows:

### 2.10 Experimental Animals

Albino rats of either sex were purchased from the National Institute of Health, Islamabad, Pakistan. They were acclimated for 1 week to regular environmental conditions (24.0°C ± 0°C temperature, 55%–65% relative humidity, 12‐h light/12‐h dark cycle) and provided formulated rodent food and water ad libitum. All the experimental protocols were approved by the Ethical Research Committee (No. DREC/20160503–18), Department of Pharmacy, University of Malakand, as per Bylaws 2008.

### 2.11 Acute Toxicity Test

An acute toxicity test of the butanol fraction was carried out by dividing the rats into various groups. The control group was fed orally with Tween‐80, while the remaining group received doses of Pe.Bt dissolved in Tween‐80 suspension, viz., 250, 500, 1000, 1500, and 2000 mg/kg body weight. The animals were thoroughly observed for any kind of behavioral and pharmacological toxicity after 1 week (Ibrar et al. 2025).

### 2.12 Induction of Diabetes

The induction of diabetes was carried out by injecting alloxan monohydrate intraperitoneally at a dose of 140 mg/kg body weight. The rats with glycosuria and hyperglycemia (higher than 220 mg/dL) after a period of 72 h of alloxan monohydrate administration were selected for this study.

### 2.13 Experimental Design

Rats were divided randomly into 6 groups in such a way that each group contained 8 rats. All were fasted overnight and the animals in Group 1 served as the normal control group, receiving normal saline. The Group 2 (diabetic control group) animals received a 5% Tween‐80 suspension. The remaining groups, i.e., Groups 3, 4, and 5, were administered Pe.Bt at doses of 150, 300, and 500 mg/kg body weight, respectively, by oral gavage. The animals in Group 6 received metformin 50 mg/kg through the same route and served as the Standard Group. The doses were administered at 09:00 am on a daily basis for 21 days. FBGL was figured out by nipping the tail (Njogu et al. 2018) using a glucometer (Accu‐check Roche Diagnostics USA) on the first day 7th, 14th, and 21st days, 2 h after the administration of various doses (Ali et al. 2015). After 24 h of the last dose administration, blood was collected from all rats via cardiac puncture under diethyl ether anesthesia for estimation of insulin level using an ELISA kit (Shah and Khan 2014) and liver function tests, followed by laparotomy for collection of pancreas and liver (Khan et al. 2017).

### 2.14 Antioxidant Activities Ex Vivo

#### 2.14.1 Estimation of Antioxidant Enzymes

After centrifuging the pancreas homogenates for an hour at 15,000× g at 4°C, the enzymes atalase (CAT) and superoxide dismutase (SOD) activity was measured. CAT activity was measured according to Beers and Sizer's protocol, which involved spectrophotometrically monitoring the reduction of hydrogen peroxide (H_2_O_2_) at 240 nm (Beers and Sizer 1952). The results were expressed in units/mg protein.

A mixture of 0.1 mL of liver homogenate and sodium carbonate buffer (2.8 mL, 0.05 mM) was incubated for 45 min at 30°C to measure SOD activity. After adding 10 μL of a 9 mM adrenaline solution, the absorbance at 480 nm was measured in comparison to a blank (Sagu et al. 1989). The units per mg of protein were used to express the results.

#### 2.14.2 Lipid Peroxidation

The lipid peroxidation inhibitory properties of Pe.BT were examined by measuring the levels of malondialdehyde (MDA)‐thiobarbituric acid reactive substances (TBARS). The formation of MDA functions as a marker for the oxidative degradation of lipids, with maximum absorption at 535 nm using a Schimadzu UV–visible spectrophotometer (Ledwoż et al. 1986).

### 2.15 Liver Function Tests

Blood was collected and centrifuged for 10 min at 1000 rpm to separate serum, which was then used to estimate alanine transaminase (ALT), aspartate aminotransferase (AST) (Moghetti et al. 2000), alkaline phosphatase (ALP) and total protein level (TP) using enzymatic kits (Vitro Scientific, Germany) according to established methods (Arman et al. 2022).

### 2.16 Determination of Liver Glycogen and Glycated Hemoglobin

A small fraction of the liver from each animal was isolated using o‐toluidine‐glucose, combined with trichloroacetic acid (TCA), separated by alcohol precipitation, and then submitted to hydrolysis. Afterwards, glycogen was quantified using a UV spectrophotometer (Swanston‐Flatt et al. 1990).

The blood samples from each rat were collected by performing a heart puncture and stored using anticoagulant agents. Glycated hemoglobin levels were measured using the procedure described by Nayak and Pattabiraman (Nayak and Pattabiraman 1981).

### 2.17 Histopathological Examination of Liver and Pancreas

The liver and pancreas were embedded in paraffin wax, sectioned with a microtome (4 μm), stained with hematoxylin and eosin (H&E), and photographed under a light microscope after being fixed in neutral buffered formalin (10%) for 24 h (Khan et al. 2017).

### 2.18 Statistical Analysis

Using GraphPad Prism Software USA, ANOVA tests were used to compare the normal control group to the control group and then the control group to that of the tested and standard tested groups. *p* values below 0.05 were deemed statistically significant. IC_50_ values were determined from dosage to dose–response curve using Microsoft Excel.

## 3 Results

### 3.1 Acute Toxicity

All the doses of Pe.Bt administered were found safe, with no mental or physical toxicity. There was no apparent change in appearance or behavior during the whole period of observation. No mortality was detected throughout the week after administration of the highest dose of 2000 mg/kg body weight.

### 3.2 In‐Vitro Anti‐Diabetic Activity

#### 3.2.1 α‐Glucosidase Assay Result

**FIGURE 1:**
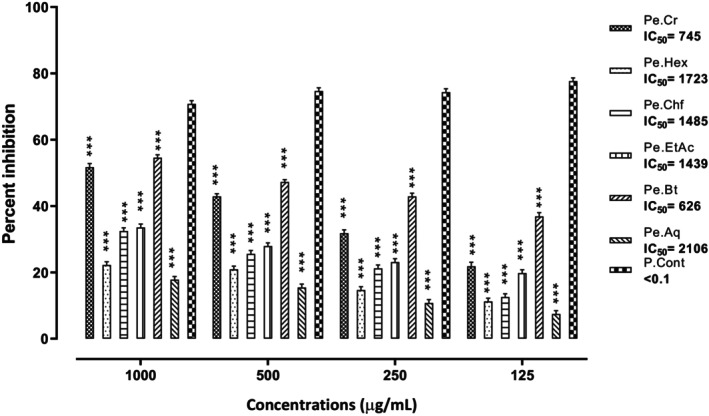
Percent α‐glucosidase inhibitory potential of various samples of *Paeonia emodi*. Each value represents mean ± SEM, *n* = 3 for each treatment group; two‐way ANOVA followed by Bonferroni test was used, all the tested samples were compared to the positive control group; ***p < 0.001.

The samples of *P. emodi* evaluated against α‐glucosidase revealed notable activities of various solvent fractions. The most active fraction of *P. emodi* was sorted out to be Pe.Bt, which exhibited 56%, 47%, 43%, and 38% α‐glucosidase inhibition at 1000, 500, 250, and 125 μg/mL, respectively. Similarly, the Pe.Cr was observed to be the second most active sample, which demonstrated 53%, 46%, 34%, and 23% enzyme inhibition at the concentrations of 1000, 500, 250, and 125 μg/mL, respectively (Figure 1). The positive control, acarbose, has an IC_50_ value of < 0.1 μg/mL, and Pe.Bt showed 626 μg/mL, which demonstrates that the activity of Pe.Bt was the highest among the plant samples. The results of various samples of *P. emodi* against α‐glucosidase have been depicted in Figure 1 as percent inhibition along with IC_50_ values.

#### 3.2.2 α‐Amylase Assay Result

**FIGURE 2:**
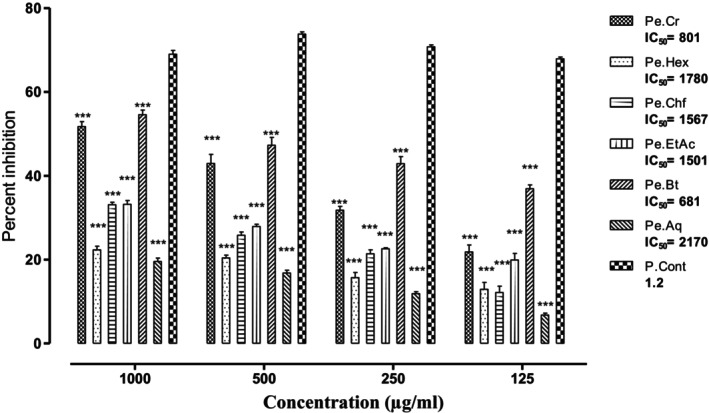
Percent α‐amylase inhibitory potential of various samples of *Paeonia emodi*. Each value represents mean ± SEM, *n* = 3 for each treatment group; two‐way ANOVA followed by Bonferroni test was used, all the tested samples were compared to the positive control group; ***p < 0.001.

The samples of *P. emodi* evaluated against α‐amylase exhibited prominent activities of various solvent fractions. The most active fraction of *P. emodi* was again sorted out to be the Pe.Bt, which exhibited 54.56%, 47.28%, 42.89%, and 36.89% inhibition against α‐amylase at 1000, 500, 250, and 125 μg/mL, respectively. Similarly, the Pe.Cr was observed to be the second most active sample, which demonstrated 51.72%, 42.92%, 31.81%, and 21.83% enzyme inhibition at concentrations of 1000, 500, 250, and 125 μg/mL, respectively (Figure 2). The positive control, acarbose, has an IC_50_ value of 1.2 μg/mL, and Pe.Bt showed 681 μg/mL, which demonstrates that the activity of Pe.Bt was the highest among the plant samples. The results of various samples of *P. emodi* against α‐amylase have been depicted in Figure 2 as percent inhibition along with IC_50_ values.

#### 3.2.3 DPP‐4 Assay Results

In the in vitro results of the tested samples against DPP4 in vitro enzymatic assay, the Pe.Bt exhibited moderate results with percent inhibition of 70.26 ± 1.96, 65.56 ± 1.76, 58.32 ± 1.52, and 52.89 ± 0.13 at concentrations of 1000, 500, 250, and 125 μg/mL, with IC_50_ value of 92.45 μg/mL. The highest activity was shown by Pe.EtAc with an IC_50_ 60 μg/mL. All the other fractions also displayed good results. The positive control ursolic acid exhibited 95.24% ± 0.90%, 91.45% ± 0.43%, 87.88% ± 0.82%, and 83.12% ± 0.14% inhibition with IC_50_ values of 7 μg/mL, respectively, as shown in Table 1.

**TABLE 1:** In vitro anti‐diabetic assay of tested samples against DPP4 and PTP‐1B.

Sample	Conc (μg/mL)	DPP4		PTP‐1B	
		% Inhibition	IC_50_ (μg/mL)	% Inhibition	IC_50_ (μg/mL)
Pe. Cr	1000	79.37 ± 1.04***		78.61 ± 0.43***	55.44
	500	72.37 ± 0.54***	62.24	72.58 ± 0.63***	
	250	65.30 ± 2.61***		65.10 ± 0.60***	
	125	58.42 ± 1.05***		59.25 ± 1.40***	
Pe. Hex	1000	56.10 ± 0.52***		74.19 ± 1.62**	150.67
	500	51.10 ± 0.56***		69.10 ± 1.10***	
	250	44.23 ± 0.13***	480.08	53.44 ± 0.42***	
	125	37.65 ± 0.37***		48.89 ± 0.19***	
Pe. Chf	1000	75.32 ± 2.87***		78.34 ± 1.16***	70.06
	500	67.12 ± 0.54***	71.08	72.88 ± 0.92***	
	250	62.79 ± 1.08***		66.67 ± 0.23***	
	125	55.79 ± 1.88***		55.78 ± 0.92***	
Pe. EtAc	1000	78.88 ± 0.89***		73.51 ± 0.54***	149.07
	500	69.54 ± 3.60***	60	65.76 ± 1.61***	
	250	65.01 ± 1.97***		57.22 ± 1.28***	
	125	57.68 ± 0.22***		47.37 ± 2.56***	
Pe. Bt	1000	70.26 ± 1.96***		83.45 ± 1.22**	45.13
	500	65.56 ± 1.76***		78.88 ± 0.22***	
	250	58.32 ± 1.52***	92.45	73.77 ± 0.11***	
	125	52.89 ± 0.13***		61.19 ± 0.13***	
Pe. Aq	1000	81.36 ± 1.49***		82.86 ± 0.86***	104.32
	500	75.34 ± 0.55***		75.52 ± 1.60***	
	250	68.39 ± 2.49**	77.06	63.02 ± 0.93***	
	125	55.44 ± 2.55***		53.66 ± 0.22***	
Ursolic acid	1000	95.24 ± 0.90			—
	500	91.45 ± 0.43			
	250	87.88 ± 0.82	7.05	—	
	125	83.12 ± 0.14			
Sitagliptin	1000			94.08 ± 1.04	21.26
	500	—	—	87.45 ± 0.90	
	250			81.58 ± 2.63	
	125			76.40 ± 3.20	

*Note:* The effect of tested compounds on AchE inhibition. The values are expressed as mean ± SEM. *p < 0.05, **p < 0.01 and ***p < 0.001 compared to standard drugs (galantamine). Data were analyzed via a two‐way ANOVA followed by a Bonferroni test.

#### 3.2.4 PTP‐1B Assay Results

In this assay, all the *P. emodi* fractions were evaluated against the PTP‐1B enzyme, which plays a significant role in diabetes. The Pe.Bt was again the most potent fraction in this assay, with percent inhibition of 83.45 ± 1.22, 78.88 ± 0.22, 73.77 ± 0.11, 61.19 ± 0.13 at concentrations ranging from 1000 to 125 μg/mL, causing IC_50_ 45.13. Likewise, the second most potent fraction was Pe.Cr with 78.61 ± 0.43, 72.58 ± 0.63, 65.10 ± 0.60, 59.25 ± 1.40, with an IC_50_ 55.44 μg/mL. All the other fractions displayed good to moderate inhibition. In this assay, sitagliptin was used as a standard drug, which exhibited excellent inhibition with an IC_50_ value of 21.26 μg/mL at the same concentration, respectively (Table 1).

### 3.3 GC–MS Analysis

The Pe.Bt fraction of *P. emodi* was subjected to GC–MS analysis to determine the presence of chemical components. Fourteen (14) main bioactive compounds in the Pe.Bt were identified and listed (Table 2). The existence of bioactive substances like octadecenoic acid, undecanoic acid, and a conjugate of exendin‐4 peptide molecules and 2 hexadecane molecules suggests possible evidence for treating diabetes, as revealed by the potency of Pe.Bt against enzymes responsible for DM.

**TABLE 2:** List of bioactive compounds present in *Pe. Bt*.

S.NO	Name	Molecular formula	Retention time
1	Isononane	C_9_H_20_	9.267
2	Trans, trans‐2,4‐Decadien‐1‐al	C_10_H_16_O	10.459
3	(2E,4E}‐2,4‐Decadienal	C_10_H_16_O	11.013
4	8‐Methy‐1‐undecene	C_12_H_24_	11.641
5	2,2,3,3‐Tetramethy Ihexane	C_10_H_22_	11.794
6	E‐2‐Tetradecen‐1‐ol	C_14_H_28_O	15.488
7	Undecanoic acid	C_11_H_22_O_2_	16.865
8	Hexadecane	C_16_H_34_	17.826
9	Tridecanoic acid, methyl ester	C_14_H_28_O	20.598
10	2‐Undecanone, 6,10‐dimethyl—	C_13_H_26_O	23.150
11	7, 9‐Di‐tert‐butyl‐1‐oxaspiro (4.5) deca‐6, 9‐diene‐2, 8‐dione	C_17_H_24_O_3_	24.679
12	9,12‐0ctadecadienoic acid, methyl ester, (E,E)—	C_19_H_34_O_2_	28.009
13	6‐0ctadecenoic acid, methyl ester, (Z)—	C_19_H_36_O	28.124
14	11‐0ctadecenoic acid, methyl ester	C_19_H_36_O	28.225

### 3.4 Antioxidant Activity

The free radical scavenging potential exhibited by various samples of *P. emodi* was noteworthy. Most importantly, the antioxidant potential of Pe.Bt against DPPH was > 50% at all concentrations, with an IC_50_ value of 77 μg/mL (Table 3). Though the positive control revealed an IC_50_ value of < 0.1, nonetheless, its percent inhibition was comparatively parallel with the Pe.Bt. All the samples were found active against DPPH at all concentrations, with a dose‐dependent response. The Pe.Chf also exhibited prominent activity against DPPH, viz., 63%, 57%, 55%, and 45% at the concentrations of 1000, 500, 250, and 125 μg/mL, respectively, with an IC_50_ value of 193. Similarly, the antioxidant effect revealed by Pe.Cr was also good, with an IC_50_ value of 367 μg/mL. The rest of the samples also displayed moderate antioxidant activity, with IC_50_ values of 1139, 763, and 1164 μg/mL for Pe.Hex, Pe.EtAc, and Pe.Aq, respectively. Furthermore, all of the *P. emodi* test samples, especially the Pe.Bt, efficiently inhibited the ABTS free radicals. Being the significant solvent fraction, the percent inhibition calculated for Pe.Bt against ABTS was recorded as 73%, 66%, 55%, and 49% at the concentrations of 1000, 500, 250, and 125 μg/mL, respectively, with IC_50_ value of 139 μg/mL. Against ABTS, the IC_50_ values recorded for Pe.Cr, Pe.Hex, Pe.Chf, Pe.EtAc, and Pe.Aq were 693, 1102, 524, 613, and 1178 μg/mL, respectively. All the tested samples of the plant extract are significantly lower than that of the standard drug ascorbic acid (***p < 0.01).

**TABLE 3:** Percent DPPH and ABTS free radical scavenging potential of various solvent fractions of *P. emodi* .

Samples	Conc (μg/mL)	DPPH		ABTS	
		Percent inhibition (mean ± SEM)	IC_50_ (μg/mL)	Percent inhibition (mean ± SEM)	IC_50_ (μg/mL)
Pe. Cr	1000	67.63 ± 0.48***		57.33 ± 0.33***	693
	500	56.00 ± 0.57***		46.46 ± 0.54***	
	250	45.03 ± 0.38***	367	35.53 ± 1.09***	
	125	34.66 ± 0.88***		34.00 ± 0.57***	
Pe. Hex	1000	46.56 ± 0.40***		47.56 ± 1.24***	1102
	500	35.50 ± 0.76***		41.76 ± 0.78***	
	250	34.96 ± 0.12***	1139	32.46 ± 0.50***	
	125	23.33 ± 0.33***		27.52 ± 0.48***	
Pe. Chf	1000	63.02 ± 1.11***		57.43 ± 0.81***	524
	500	57.10 ± 0.91***		49.12 ± 0.88***	
	250	55.83 ± 0.88***	193	46.56 ± 1.24***	
	125	45.07 ± 1.02***		35.16 ± 0.86***	
Pe. EtAc	1000	57.26 ± 0.52***		56.53 ± 1.09***	613
	500	46.70 ± 0.69***		47.63 ± 1.78***	
	250	45.63 ± 0.27***	763	45.46 ± 0.68***	
	125	34.00 ± 1.06***		37.06 ± 1.04***	
Pe. Bt	1000	71.03 ± 0.87***		73.33 ± 0.88***	139
	500	66.66 ± 0.66***		66.00 ± 0.57***	
	250	59.83 ± 0.29***	77	55.50 ± 0.60***	
	125	54.16 ± 0.85***		49.16 ± 0.60***	
Pe. Aq	1000	45.43 ± 0.61***		42.00 ± 0.57***	1178
	500	34.49 ± 0.84***		32.02 ± 1.11***	
	250	29.63 ± 0.52***	1164	24.10 ± 0.58***	
	125	23.26 ± 0.32***		21.66 ± 0.49***	
Ascorbic acid	1000	89.00 ± 0.57		85.00 ± 0.57	0.470
	500	88.16 ± 0.85		81.10 ± 0.58	
	250	84.63 ± 0.52	1.42	79.33 ± 0.33	
	125	80.02 ± 1.11		77.33 ± 0.88	

*Note:* All values are represented as Mean ± SEM (*n* = 3), Values significantly different in comparison to standard drug i.e., ***p < 0.001. Data were analyzed via TWO‐WAY ANOVA followed by Bonferroni test.

### 3.5 Anti‐Diabetic Potential *(*In Vivo*)*

**FIGURE 3:**
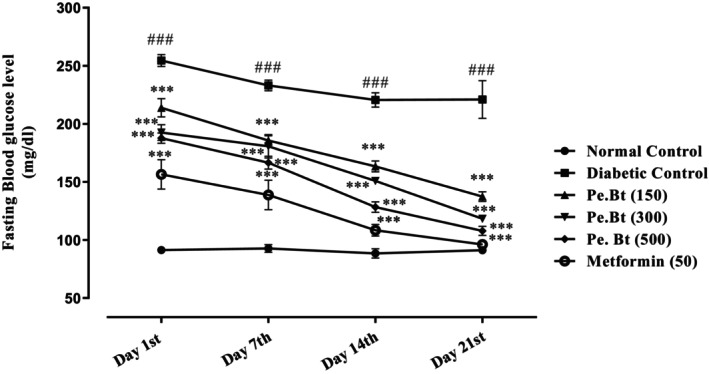
In vivo anti‐diabetic activity of butanol fraction of *Paeonia emodi* in alloxan‐induced fasting diabetic rats. Each value represents mean ± SEM, *n* = 6 for each treatment group; RM‐TWO‐WAY ANOVA followed by Bonferroni test was used, all the test samples were compared to the diabetic control; ***p < 0.001, while diabetic control groups were compared to the normal control group; ^###^ *p* < 0.001.

Based on the prominent in vitro antidiabetic and antioxidant activities, Pe.Bt was evaluated for in vivo antidiabetic potential at a dose of 150, 300, and 500 mg/kg body weight of the rat. The results are summarized in Figure 3, representing normal control, diabetic control, various concentrations of Pe.Bt, and standard group. As this activity was recorded for 3 weeks, it is obvious that on day 1, the decrease in FBGL was significant for metformin, followed by 500, 300, and 150 mg/kg of Pe.Bt in a dose‐dependent manner (***p < 0.01). Likewise, on day 7, the diabetic control has a value of 233 mg/dL, which is smaller than that of day 1, and the same is the case with the doses of Pe.Bt, which decreased the FBGL from 233 to 166 mg/dL by 500 mg/kg (***p < 0.01). On the 14th day, the results of metformin and the highest dose of Pe.Bt were somewhat comparable, exhibiting FBGL of 108 (*p* < 0.01) and 128 mg/dL (***p < 0.01) respectively. The results on day 21 showed **a** significant decrease in FBGL. The result of normal control and metformin was almost the same, i.e., 91 and 96 mg/dL, respectively. In the same way, the highest dose of Pe.Bt also displayed a significant decrease in FBGL, i.e., 108 mg/dL (***p < 0.01), Figure 3.

### 3.6 Serum Insulin Level

**FIGURE 4:**
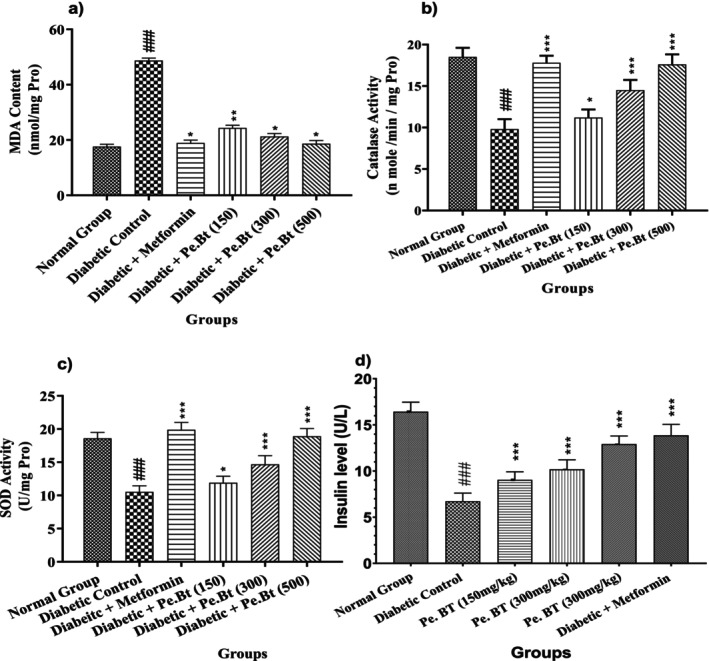
(a–d): Estimation of antioxidant enzymes in vivo and insulin level. Each value represents mean ± SEM, *n* = 6 for each treatment group; Two‐way ANOVA followed by Bonferroni test was used in this assay, and all the test samples were compared to the diabetic control; ***p < 0.001, **p < 0.01, *p < 0.05 while diabetic control groups were compared to the normal control group; ^###^ *p* < 0.001.

The effect of *Pe.Bt* extract at different concentrations on serum insulin levels in diabetic rats was investigated on the 21st day and compared with the standard drug metformin. The induction of diabetes significantly decreased insulin levels in the diabetic control group (6.73 ± 0.88 μU/L) compared to the normal control group (16.43 ± 1.03 μU/L), as shown in Figure 4d. Treatment with *Pe.Bt* extract exhibited a dose‐dependent increase in insulin levels. At doses of 150, 300, and 500 mg/kg, insulin levels increased to 9.04 ± 0.88, 10.19 ± 1.03, and 12.92 ± 0.88 μU/L, respectively. The highest dose (500 mg/kg) of *Pe.Bt* markedly improved insulin secretion, nearly the effect produced by metformin (13.87 ± 1.18 μU/L).

### 3.7 Estimation of Antioxidant Enzymes *(*In Vivo*)*

The liver tissue homogenate was used to measure the CAT and SOD activity. When compared to the normal control group, the administration of alloxane significantly lowered the levels of CAT and SOD activities in the liver tissue. On the other hand, treatment with 150, 300, and 500 mg/kg body weight of Pe.BT increased this enzyme activity in a dose‐dependent manner. In particular, CAT and SOD activities were considerably higher in the animals pretreated with Pe.BT at a dose of 500 mg/kg, at 17.6 ± 1.22 nmole/min/ml proteins and 18.9 ± 1.18 units/mg proteins, respectively, than in the alloxane‐treated group, as shown in Figure 4b,c.

### 3.8 Lipid Peroxidation

MDA levels in the homogenate were measured to determine the degree of lipid peroxidation in the liver tissues, as indicated in Figure 4a. The liver tissues of the control group showed a modest amount of MDA (17.6 ± 0.88 nmol/mg protein). On the other hand, the MDA level (48.70 ± 0.92 nmol/mg protein) in the diabetic control group was considerably higher, suggesting enhanced lipid peroxidation. The study groups exhibited the dose‐dependent impact of Pe.BT on averting lipid peroxidation in animals treated with alloxan. A moderate preventative effect was indicated by the MDA level of 24.35 ± 0.98 nmol/mg protein in the group treated with 150 mg/kg of Pe.EA. When the dose of Pe.BT was increased to 300 mg/kg, the MDA level was further decreased to 21.28 ± 1.02 nmol/mg protein. Pe.BT significantly improved the prevention of lipid peroxidation at a dose of 500 mg/kg, bringing the MDA level down to 18.70 ± 1.12 nmol/mg protein, which was similar to the control group.

### 3.9 Liver Function Tests

**FIGURE 5:**
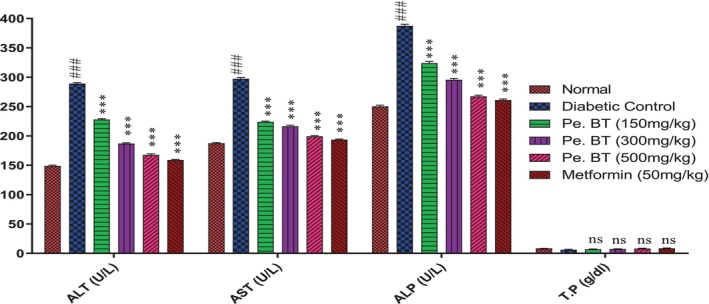
Effect of PE.Bt on liver function tests in Alloxan‐induced diabetic rats. Each value represents mean ± SEM, *n* = 6 for each treatment group; independent one‐way ANOVA was used in this assay, and all the test samples were compared to the diabetic control; ***p < 0.001, while diabetic control groups were compared to the normal control group; ^###^ *p* < 0.001.

In rats treated with alloxan, the serum levels of ALT, AST, and ALP were all considerably increased, and the serum level of TP decreased by 2.70 g/dL. Animals treated with Pe.BT 150 mg/kg reduced the levels of ALT, AST, and ALP to 68.74 ± 1.06, 83.85 ± 1.33, 223.98 ± 3.02 U/L and decreased the serum level of TP to 06.03 ± 0.73 g/dL. Comparing the Pe.BT 500 mg/kg group to the alloxan‐treated group, there was a substantial decrease in the levels of ALT, AST, and ALP. It reduced the ALT level from 289.15 ± 1.73 U/L (***p < 0.001) to 167.58 ± 1.86 U/L (**p < 0.01) as compared to metformin Figure 5.

### 3.10 Effect on Liver Glycogen Levels and Glycosylated Hemoglobin

**FIGURE 6:**
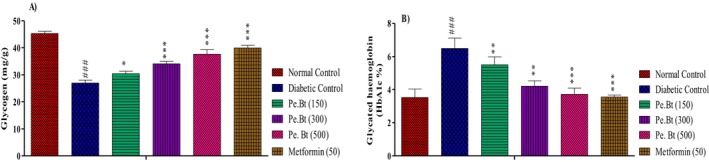
Effect on liver glycogen levels and glycocylated hemoglobin after respective treatment. Each value represents mean ± SEM, *n* = 6 for each treatment group; two‐way ANOVA followed by Bonferroni test was used in this assay. All the tested samples were compared to the diabetic control; ***p < 0.001, **p < 0.01, *p < 0.05 while diabetic control groups were compared to the normal control group; ^###^ *p* < 0.001.

The amounts of hepatic glycogen and glycosylated hemoglobin in the experimental animals are shown in Figure 6. The animals in the Diabetic Group showed lower levels of hepatic glycogen and higher levels of glycosylated hemoglobin, at 26.92 ± 1.12 mg/g and 6.50% ± 0.62% respectively. After receiving the Pe.Bt in different doses, i.e., 150, 300 and 500 mg/kg, diabetic rats' levels of glycosylated hemoglobin decreased along with an increase in hepatic glycogen stores. Pe.Bt at a dose of 500 mg/kg significantly reduced the levels of glycosylated hemoglobin to 3.72% ± 0.38% and increased the levels of glycogen to 37.63 ± 1.73 mg/g.

### 3.11 Histopathological Examination of the Liver and Pancreas

**FIGURE 7:**
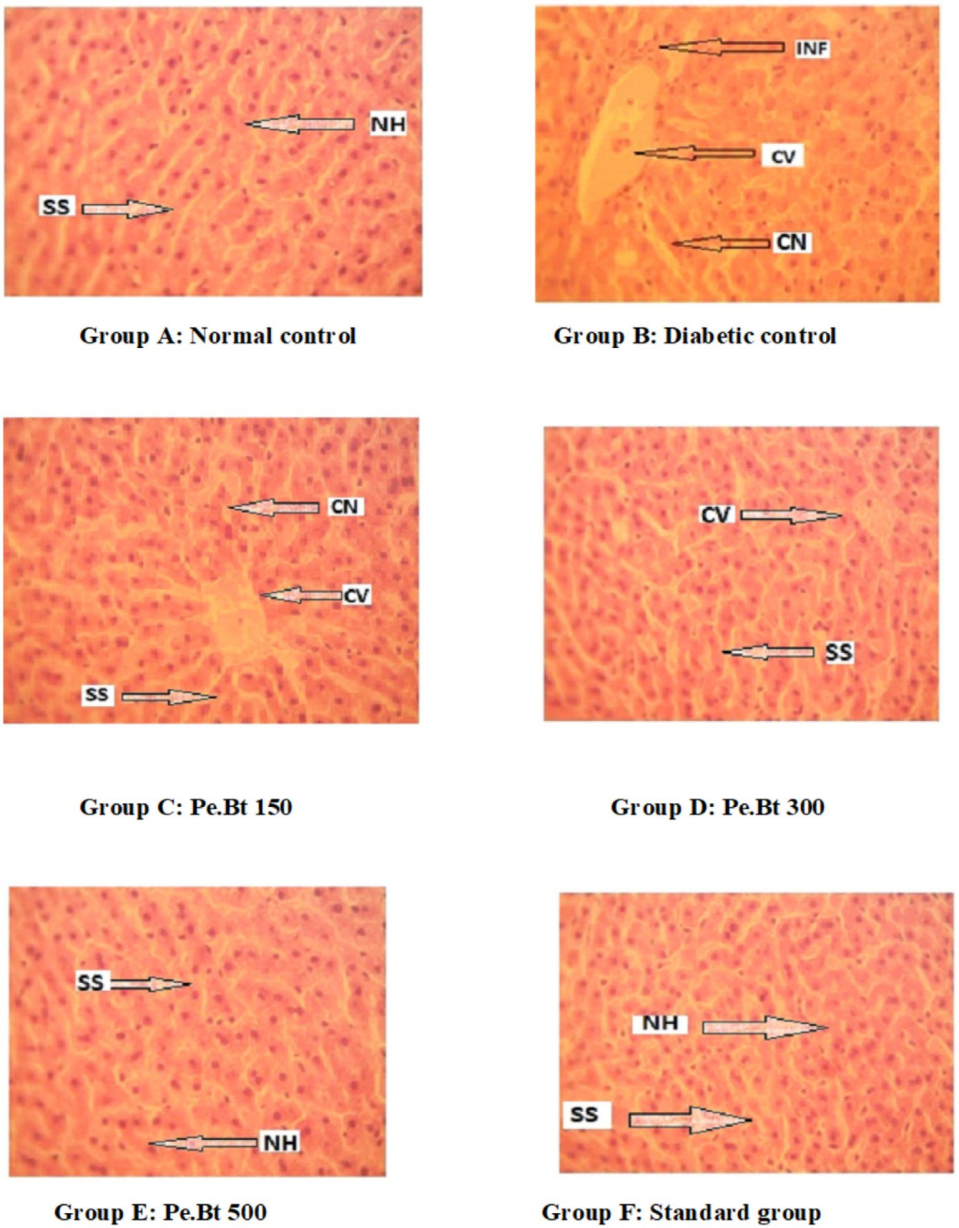
Photomicrograph of the liver of rats stained with hematoxylin and eosin ×40. CN, cellular necrosis; CV, central venule; HN, hepatocellular necrosis; INF, cellular infiltration; NH, normal hepatocytes; SS, sinosiods.

**FIGURE 8:**
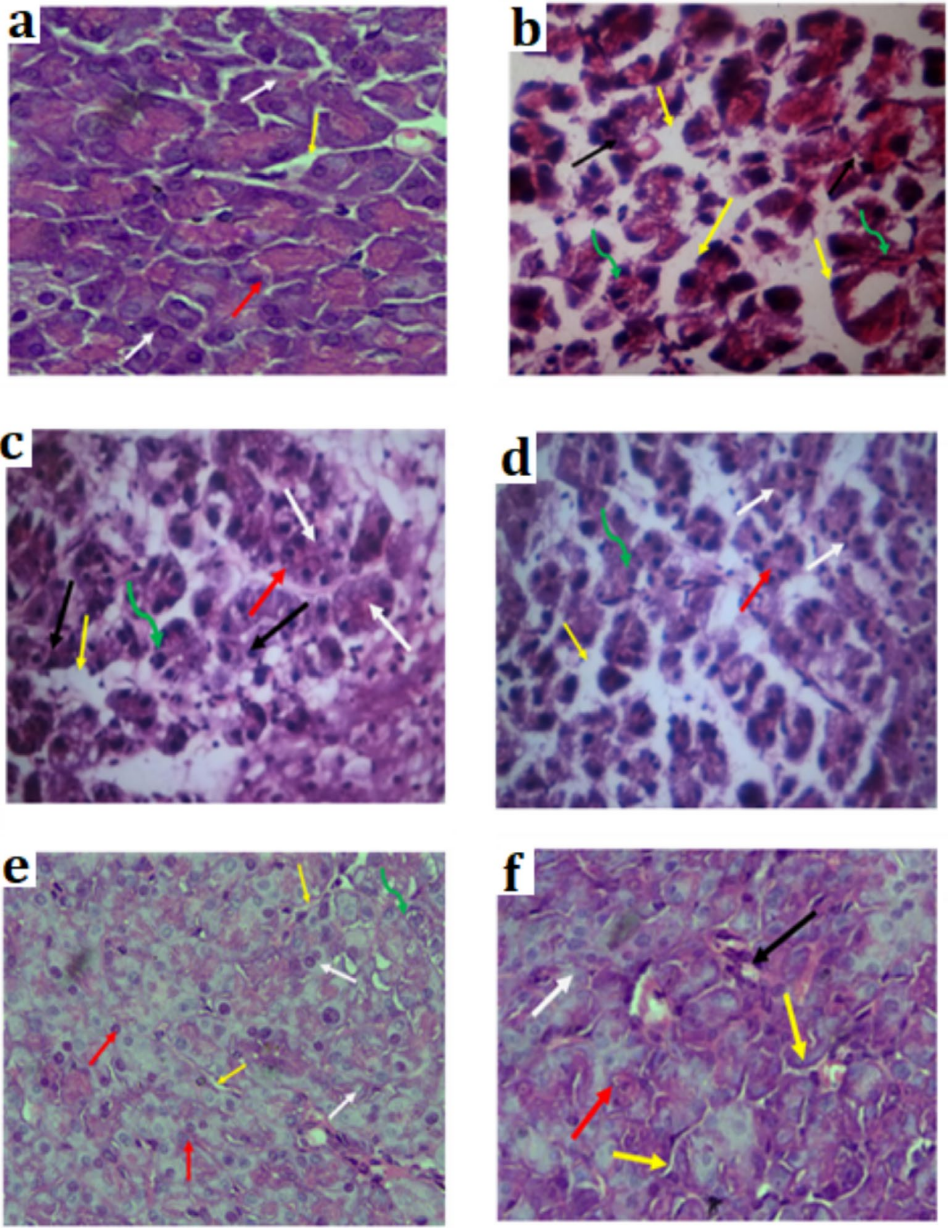
Histopathological changes of pancreatic tissues of (a) control group, (b) diabetic control, (c) Pe.BT 150 mg/kg, (d) Pe.BT 300 mg/kg, (e) Pe.BT 500 mg/kg, and (f) standard group. White arrow: Normal pancreatic islets, red arrow: Normal pancreatic acini, yellow: Interlobular connective tissues, green curve arrow: Degenerative changes in nucleus of acini, black arrow: Abnormal islets.

To investigate whether the Pe.BT treatments at three different doses had any impact on the alloxan‐induced diabetic liver tissues, a histopathological examination was carried out. In the normal control group (Figure 7, Group A), the histological structure of the liver was observed to be normal. The livers of the diabetic rats in Figure 7, Group B, are completely devoid of hepatocytes and are characterized by severe congestion, lobule loss, and congested hepatic inflammation. The arrangement of hepatocytes in the livers of rats administered three different doses (150, 300 and 500 mg/kg) of Pe.BT and rats treated with metformin (50 mg/kg)closely resembled the normal control group. Similarly, the histopathological investigations of pancreatic tissues of different groups are represented in Figure 8 as follows. There were no pathological alterations in the normal control group animals' pancreatic tissue architecture (Figure 8a). However, the alloxan diabetic rats' pancreatic tissues showed severe histopathological damage, including a complete loss of islet cells and an increase in acinar cells (Figure 8b). The standard group did not show any marked variations (Figure 8f). In comparison to the standard and normal groups, the histological examination of the pancreatic tissues of the test dose 150 mg/kg exhibited substantial variance, such as inflammatory infiltration and degenerative changes in the nucleus of acini (Figure 8c). Only minor degenerative changes were observed in the pancreatic tissues at 300 mg/kg (Figure 8d), suggesting islet‐cell regeneration and a return to normal histology. In contrast, a test dose of 500 mg/kg (Figure 8e) demonstrated significant islet cell regeneration, fewer degenerative changes in the nucleus of acini, and abnormal islets.

## 4 Discussion

DM is considered one of the most threatening diseases to human health all over the world, characterized by high blood glucose levels due to destruction of islet cells, which leads to less production of insulin. DM may be fatal in some cases if not treated properly (Kaliaperumal et al. 2024; Altas et al. 2011). Various treatment approaches are used to treat DM, but the antidiabetic drugs available in the market cause side effects and increase drug resistance in patients with DM (Li et al. 2013). Hence, it is necessary to explore a conventional medication therapy having fewer side effects with strong antidiabetic potential. Before the discovery of insulin, various plants were traditionally used for the treatment of DM, and they are still in practice because of their easy availability, safety, and fewer side effects (Osadebe et al. 2014). In the current study, PE.BT was validated for its antidiabetic potential through in vitro and in vivo investigations in Alloxan‐induced DM in rats. This is strongly evident from the previously reported data that free radicals can cause lipid peroxidation, which can initiate a cascade of biochemical reactions (Buettner 1993). This may lead to the inactivation of various enzymes, the glycation of proteins, alteration of physiology, and structure of the basement membrane and collagen, thereby instigating complications of DM (Wolff 1993; Sabu and Kuttan 2004). In our current investigational study, we have exploited alloxan for the induction of DM, which has been induced due to the development of oxidative stress, as evident from the previously reported literature (Sabu and Kuttan 2004). This emphasizes that alleviation of oxidation stress and scavenging of free radicals can help a diabetic patient fight against diabetes and its complications. In another way, the enzyme responsible for the breakdown of saccharides and the production of glucose units, that is, α‐glucosidase, is also known to be effectively involved in the increase of the blood glucose level, which has been used as a successful target to decrease the absorption of glucose from the gastrointestinal tract (Ag 1994). Numerous synthetic and naturally occurring bioactive substances have been shown to possess potent α‐glucosidase inhibitory capabilities (Wysowski et al. 2003; Asano et al. 2000). It is obvious from Table 1 that *P. emodi* possesses a potent inhibitory effect against PTB‐1B and DPP‐4 enzymes responsible for DM. The enzymatic inhibition mechanism of Pe.BT demonstrates a strong inhibitory effect on α‐glucosidase and α‐amylase (Figures 1 and 2), indicating its potential role in preventing postprandial hyperglycemia (Ajiboye et al. 2016). The phytochemical screening of *P. emodi* revealed a diverse array of bioactive compounds, including fatty acid esters, alkenes, aldehydes, and ketones, many of which have been reported to exhibit pharmacological activities relevant to diabetes management. Notably, compounds such as 9,12‐octadecadienoic acid (linoleic acid) methyl ester and 11‐octadecenoic acid methyl ester (oleic acid derivative) are known for their antioxidant, anti‐inflammatory, and insulin‐sensitizing properties. The presence of aldehydes like (2E,4E)‐2,4‐decadienal and trans,trans‐2,4‐decadien‐1‐al, which may influence glucose metabolism, further supports the potential of *P. emodi* as a source of antidiabetic agents. Additionally, undecanoic acid and tridecanoic acid methyl ester are medium‐chain fatty acid derivatives that may contribute to improved lipid metabolism. These findings highlight the therapeutic promise of the extract and provide a biochemical rationale for its observed acute antihyperglycemic effect (Shafie et al. 2015). Undecanoic acid has been previously reported to inhibit the activity of DPP‐4 (Tasyurek et al. 2014). Inhibition of DPP‐4 activity specifically results in higher glucagon‐like peptide‐1 levels, and increases the half‐life of circulating insulin, thereby enhancing glycemic control in individuals with type 2 diabetes (Tasyurek et al. 2014). Similarly, a conjugate prepared that consists of two exendin‐4 peptide molecules and 2 hexadecane molecules has been reported for the treatment of diabetes using 4‐arm PEG20K as the linker (Kim et al. 2011). Referring to Figure 3, this becomes clear that the in vivo anti‐diabetic effect of the plant sample was absolutely prominent on the 21st day of observation, with no mortality or morbidity seen, suggesting that Pe. BT effectively alleviates the symptoms of diabetes within a time frame of 3 weeks and can be utilized safely with no noxious effect (Lucchesi et al. 2015). The alloxan‐induced diabetic animals are commonly utilized due to their reliability and ability to consistently produce a hyperglycemic state. However, this model closely mimics the characteristics of Type 1 diabetes, as alloxan selectively damages pancreatic β‐cells through oxidative stress, leading to significant insulin deficiency. It does not replicate the insulin resistance and progressive β‐cell dysfunction that define Type 2 diabetes, which is the predominant form of diabetes worldwide. Considering that the introduction of this study emphasizes the growing global prevalence of diabetes, which was driven largely by the rise in Type 2 cases. This is the limitation of the current model which was used. Although the alloxan model offers valuable insights into hyperglycemia and β‐cell‐related interventions, its ability to reflect the complex metabolic disturbances involved in Type 2 diabetes, including insulin resistance, systemic inflammation, and lipid metabolism disorders, is limited. As such, the findings of this study should be interpreted with caution, bearing in mind the specific pathophysiological context of the model used. For broader relevance, future research should incorporate experimental models that more closely represent Type 2 diabetes, such as high‐fat diet combined with low‐dose streptozotocin (STZ), or genetic models like the db/db and ob/ob mice. These approaches would help validate and extend our findings in a manner more applicable to the clinical spectrum of Type 2 diabetes. A histological study of selected livers and pancreas from both Alloxan‐treated and untreated rats revealed morphological abnormalities and microscopic alterations. The pathological changes in the pancreatic exocrine and endocrine tissues were shown by the histological analysis of pancreatic segments. The development of insulin resistance is followed by a severe lesion characterized by the loss of 𝛽‐cells and the degeneration of the islets of Langerhans (Yousef et al. 2022). When rats were given 150 mg/kg, their pancreatic tissues displayed noticeable inflammation or infiltration; nevertheless, the tissue architecture significantly improved at 500 mg/kg dose and resulted in normalization of the islets of Langerhans. The Pe.BT at the highest dose protected the histological alterations in the diabetic rat's pancreas, which suggests the protective and curative effect on the pancreas, restoring the biochemical balance, reducing the extent of damage in alloxan‐induced diabetic rats as produced by the standard drug (Figures 7 and 8).

*Paeonia emodi* has been previously reported to have numerous bioactive compounds (Riaz et al. 2003). So, the anti‐diabetic and antioxidant potential of *P. emodi* may be due to the presence of a wide variety of secondary metabolites. Moreover, the presence of some important medicinal components, i.e., secondary metabolites, has also been reported and isolated previously from *P. emodi* with diverse pharmacological activities, which further testifies to the anti‐diabetic potential of this plant (Riaz et al. 2004; Zargar et al. 2014).

## 5 Conclusion

In the current investigational study, the anti‐diabetic activity of *P emodi* has been verified with various possible mechanisms. So, based on the recorded data and previous reports, it may be inferred that *P. emodi* contains bioactive compounds with hepatoprotective and anti‐diabetic properties. It may also be concluded that this plant can be used safely by the disadvantaged community to avoid diabetes mellitus. This study should be further narrowed down to the isolation of active compounds, which may lead to novel drug development.

## Author Contributions

**Jirui Fan:** writing – review and editing (equal). **Muhammad Ibrar:** conceptualization (equal) investigation (equal). **Mir Azam Khan:** supervision (equal). validation (equal). **Abdullah:** data curation (equal). **Muhammad Saeed Jan:** investigation (equal). **Madeeha Shabnam:** data curation (equal). **Maqsood ur Rehman:** conceptualization (equal).

## Ethics Statement

All the experiments in this manuscript were approved by the Department of Pharmacy, University of Malakand, departmental ethical committee with ethical approval number DREC/2022098/06.

## Consent

We declare that the Publisher has the Author's permission to publish the relevant contribution.

## Conflicts of Interest

The authors declare no conflicts of interest.

## Data Availability

The data will be provided with the permission of corresponding authors.
