# Correction to “Phytochemical Screening and Antidiabetic Potential of *Paeonia emodi* in Alloxan‐Induced Diabetic Rats: Investigative Study Toward Possible Mechanism”

**DOI:** 10.1002/fsn3.71033

**Published:** 2025-09-26

Fan, J., M. Ibrar, M. A. Khan, et al. 2025. “Phytochemical Screening and Antidiabetic Potential of *Paeonia emodi* in Alloxan‐Induced Diabetic Rats: Investigative Study Toward Possible Mechanism.” *Food Science & Nutrition* 13, no. 9: e70870. https://doi.org/10.1002/fsn3.70870.

In section 3.9. “Liver function tests,” the sentence “Animals treated with Pe.BT 150 mg/kg reduced the levels of ALT, AST, and ALP to 68.74 ± 1.06, 83.85 ± 1.33, 223.98 ± 3.02 U/L and decreased the serum level of TP to 06.03 ± 0.73 g/dL.” was incorrect; this should have read as “Animals treated with Pe.BT 150 mg/kg reduced the levels of ALT, AST, and ALP to 228 ± 1.06 U/L, 223.85 ± 1.33 U/L, and 323.98 ± 3.02 U/L and decreased the serum level of TP to 06.92 ± 0.64 g/dl.”

We apologize for these errors.

